# Diversity of Rotavirus Strains Causing Diarrhea in <5 Years Old Chinese Children: A Systematic Review

**DOI:** 10.1371/journal.pone.0084699

**Published:** 2014-01-08

**Authors:** Yue Li, Song-Mei Wang, Shan-Shan Zhen, Ying Chen, Wei Deng, Paul E. Kilgore, Xuan-Yi Wang

**Affiliations:** 1 Key Laboratory Medical Molecular Virology, MoE/MoH, and the Institutes of Biomedical Sciences, Shanghai Medical College, Fudan University, Shanghai, People's Republic of China; 2 Training Center of Medical Experiments, Shanghai Medical College, Fudan University, Shanghai, People's Republic of China; 3 Department of Health Statistics & Social Medicine, School of Public Health, Shanghai Medical College, Fudan University, Shanghai, People's Republic of China; 4 Eugene Applebaum College of Pharmacy and Health Sciences, Wayne State University, Detroit, United States of America; Centers for Disease Control and Prevention, United States of America

## Abstract

**Background:**

We conducted a systematic review of the diversity and fluctuation of group A rotavirus strains circulating in China.

**Methods and Findings:**

Studies of rotavirus-based diarrhea among children less than 5 years, published in English or Chinese between 1994 and 2012, were searched in PubMed, SinoMed, and CNKI and reviewed by applying standardized algorithms. The temporal and spatial trends of genotyping and serotyping were analyzed using a random-effects model. Ninety-three studies met the inclusion/exclusion criteria and were included in the meta-analysis. Overall, 22,112 and 10,660 rotavirus samples had been examined for G and P types, respectively. The most common G types were G1 (39·5%), G3 (35·6%), G2 (1·3%), and G9 (0·1%). Among P types, P[8] (54·6%) was the predominant type, followed by P[4] (11·1%) and P6 (0·1%). The most common G-P combinations were G3P[8] (32·1%) and G1P[8] (24·5%), followed by G2P[6] (13·2%) and G2P[4] (10·1%). Before 2000, serotype G1 was the predominant strain and accounted for 74·3% of all rotavirus infections; however, since 2000, G3 (45·2%) has been the predominant strain. Rotavirus P types showed little variation over the study period.

**Conclusion:**

Despite the variation of serotypes observed in China, the G1, G2, G3, and G4 serotypes accounted for most rotavirus strains in recent decades. These results suggest that Chinese children will be adequately protected with currently available or forthcoming rotavirus vaccines.

## Introduction

Acute gastroenteritis is a leading cause of childhood illness in China [1], [2]. Rotavirus is the pathogen most commonly identified as causing severe gastroenteritis in children less than 5 years old. In China, published data show that rotavirus-associated hospitalizations account for 32% to 50% of all hospitalizations for diarrhea among infants and children <5 years of age [3]–[8]. Beside of the current rotavirus vaccine (ie, the Lanzhou lamb rotavirus vaccine, LLR), which was developed and licensed in China in 2000, Two reassortant vaccines are being developed in China, one of which is based on the UK-bovine reassortant rotavirus vaccine originally developed at the National Institutes of Health (USA). To successfully develop and introduce a rotavirus vaccine in China, a full picture on the distribution of rotavirus G and P types is needed.

## Methods

### Search strategy

We identified studies of diarrhea caused by rotavirus among children less than 5 years old in mainland China, published before June, 2012, using standardized search algorithms for systematic reviews [9]. Scientific articles published in English or Chinese were identified using PubMed (United States), SinoMed (Chinese Bio-Medical Literature Service System, China), and CNKI (National Knowledge Infrastructure, China) databases. Standardized medical subject heading (MeSH) term “rotavirus” and free word “China” were used to search each database.

### Definitions

To facilitate identification of reports, a suspected case of rotavirus diarrhea was defined as a child less than 5 years old who was admitted for treatment of diarrhea to a healthcare facility. A confirmed case of rotavirus diarrhea was defined as a child for whom rotavirus infection was proven by means of an enzyme immunoassay performed on fecal specimens.

### Review strategy

Endnote® (version X, Thomson Reuters, Inc., Philadelphia, PA) bibliographic software was used to create an electronic library of citations identified in the database searches. PubMed searches were performed using Endnote®, and duplicate records were deleted. Each study was assigned a unique identification code to enable tracking of reviews and analysis after title/abstract screening. Two independent reviewers were trained to perform the title/abstract screening and thereafter full text screening. Disagreements were resolved by consensus between the two reviewers and the corresponding author. For each article that met the inclusion criteria we used a structured questionnaire to appraise its quality based on study design, sources of specimens, study scope, case definitions, and diagnostic methods. To identify differences in homogeneity among the articles, each question was assigned a score, with zero being the lowest and five being the highest. Mean points per question were calculated for each article. Articles with ≥3·0 points/question were considered moderate/high quality. A structured questionnaire was used for data extraction, and the EpiData program (version 3·1) was used for data entry.

A pilot study was conducted to refine the questionnaire for quality assessment, and inclusion/exclusion criteria. The principles of Transparent Reporting of Systematic Reviews and Meta-Analysis (PRISMA) were implemented throughout the study.

### Study inclusion and exclusion criteria

All studies published before June, 2012, in English or Chinese languages, obtained from the sources defined above, were considered for inclusion. To be included, published reports possessed the following characteristics: 1) the study population was composed exclusively of children less than 5 years old living in mainland China; 2) group A rotavirus strains were reported as the etiologic agent; 3) results reported data on rotavirus G and P types; and 4) the strains used for G and P typing numbered at least 50. Reports were excluded from final analysis if their content was limited to a description of diagnostic methods, traditional medical therapies, case reports, health education, or policy analysis or if they focused solely on reporting community outbreaks and nosocomial cases, as these data could bias the distribution of serotypes or genotypes found in annual endemic transmission.

### Analytical strategy and statistical analysis

The temporal and spatial distributions of rotavirus G and P types were analyzed to describe changes over time and by region within China. Differences among studies in the use of an enzyme immunoassay (EIA) or polymerase chain reaction (PCR)-based test for rotavirus were considered when appraising study quality, but test results using either method were pooled for the meta-analysis. To study temporal variations in rotavirus strain types, the extracted data were grouped by the year the study was published, namely 1994–1999, and 2000–2012, considering the implementation of LLR vaccine in 2000 in China. The Qinling Mountain-Huaihe River line, which is commonly recognized as the north-south dividing line of mainland China [10], was applied to define the geographic regions, namely South (subtropical zone) and North (warm temperate zone).

Before the original data were synthesized, homogeneity across studies was tested by use of the Q statistic (*P*<0·10)[11]. If substantial heterogeneity existed, the random-effects model was used; otherwise, the fixed-effects model is preferred to summarize the pooled percentage, as well as 95% confidence intervals [12]. In order to detect potential bias introduced by the heterogeneous quality of publications, the pooled percentages from all studies and high-quality articles (mean score per question ≥3·0) were calculated and compared. If there was significant difference, outputs derived from high-quality articles would be presented. All analyses were performed using STATA version 10·0 (Stata Corp, College Station, Texas, USA). Statistical significance was assessed using *P*<0·05.

### Ethics

This study was reviewed and approved by the Institutes of Biomedical Sciences Institutional Review Board, Fudan University.

## Results

### Overview of studies

**Figure 1:**
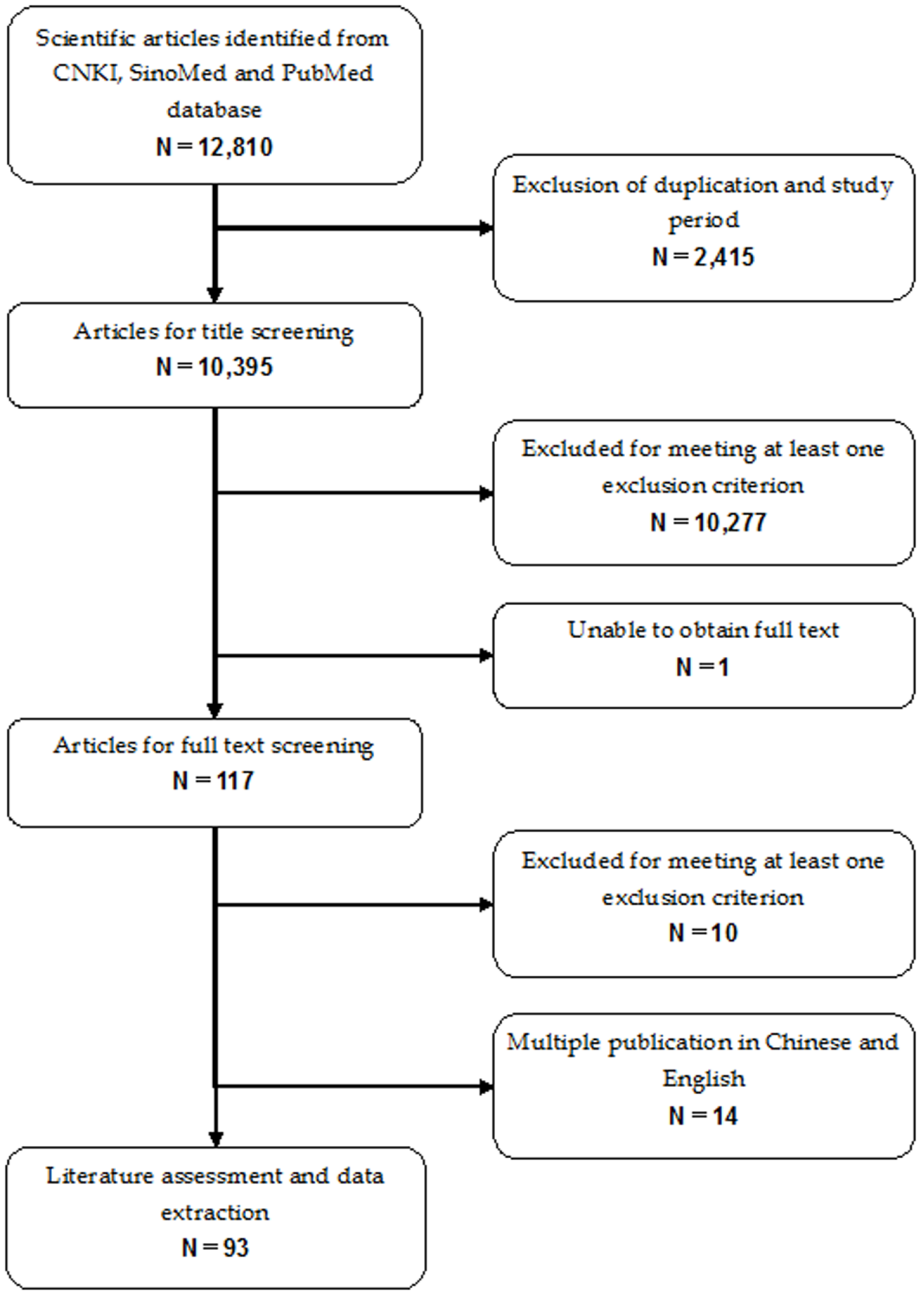
Eligibility of studies for inclusion in this systematic review. The initial search identified 12,810 rotavirus-related citations from CNKI, SinoMed, and PubMed. A total of 93 studies that met the inclusion and exclusion criteria were included in the pooled analysis.

**Figure 2:**
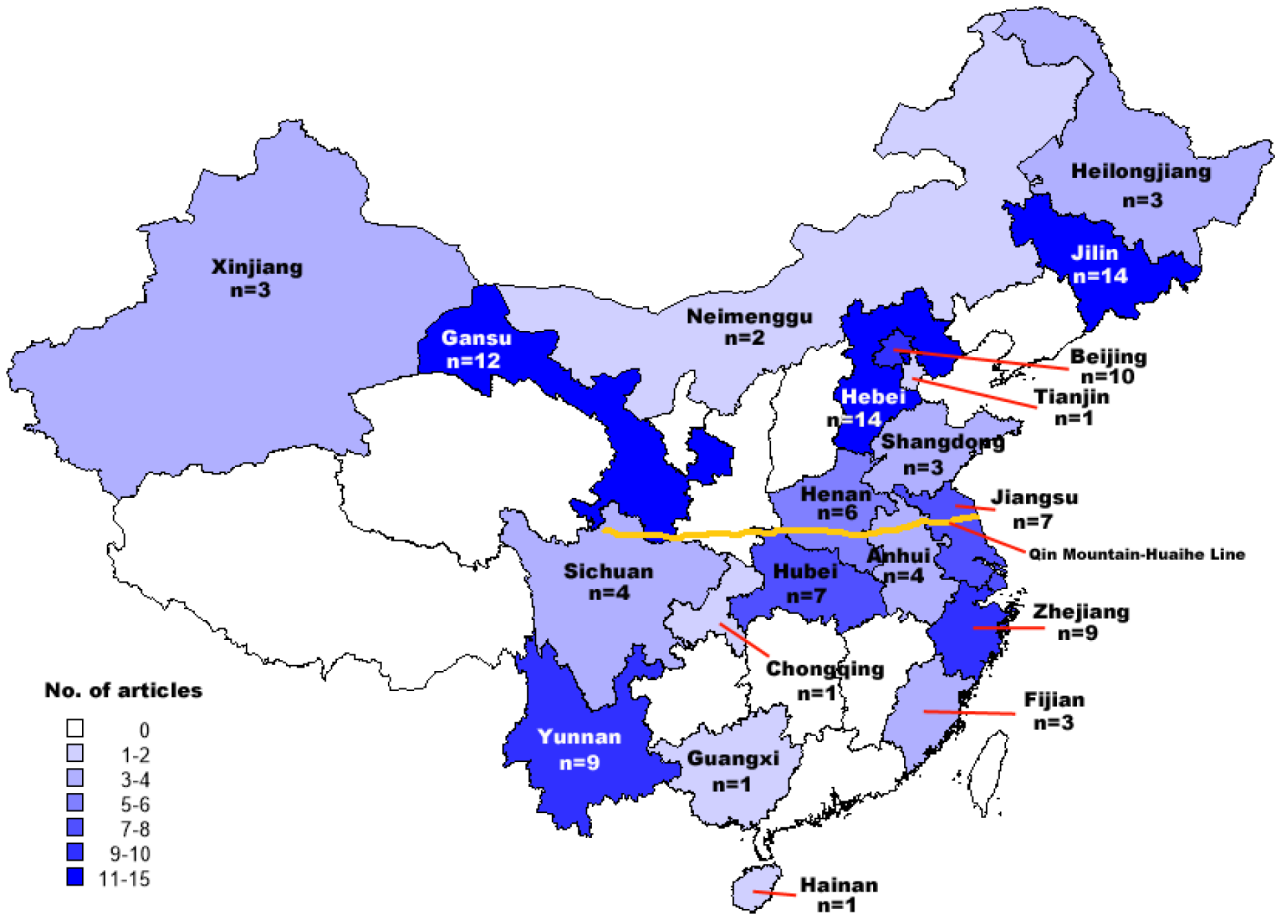
Distribution of included articles by province. The 93 studies included in the pooled analysis came from 22 provinces.

The initial search identified 12,810 rotavirus-related citations from CNKI, SinoMed, and PubMed. After first excluding duplicate records, 10,277 and 10 studies were excluded after review of title/abstract and full text information, respectively (**Figure 1**). A total of 93 studies (83 Chinese articles and 10 English articles) that met the inclusion and exclusion criteria were included in the pooled analysis. Of these, 24 (25·8%) studies were appraised as moderate/high quality, with a mean score ≥3·0 per question. Approximately 70% of specimens were collected from inpatients treated in city- or county-level hospitals. The majority of studies were conducted in eastern provinces of China (n = 51, 54·8%). Other studies were identified from central (n = 33, 35·5%) and western provinces (n = 27, 29·0%) (**Figure 2**). Regarding the G- and P-typing, most studies (>90%) tested only for common G and P types, namely G1–G4, G9 as well as P[4], P[6], P[8], P[9], and P[10]. Out of 93 studies included in final analysis, only 8 studies (8·6%) reported rotavirus G and P types identified by nucleic acid sequencing (**Table 1**).

**Table 1:** Characteristics of all studies included in the meta-analysis (n = 93).

Characteristic	Studies (n)	%
Journal		
International English	10	10·8
Domestic Chinese core	43	46·2
Domestic Chinese non-core	40	43·0
Study region*		
Eastern	51	54·8
Central	33	35·5
Western	27	29·0
Source of specimens		
Outpatient	7	7·5
Inpatient	65	69·9
Both	21	22·6
Study scope		
Nation	10	10·8
Province	17	18·2
City/county	66	71·0
Typing confirmed by sequencing		
Yes	8	8·6
No	85	91·4
Case inclusion criteria		
Presented	40	43·0
Not presented	53	57·0
Diagnostic method for RV infection		
ELISA?	77	82·8
PAGE$	16	17·2
Mean score after quality assessment		
<2·5	28	30·1
2·5 -	41	44·1
3·0-	16	17·2
≥3·5	8	8·6

?: Abbreviations: ELISA =  enzyme-linked immunosorbent assay;

^$^: Abbreviations: PAGE =  polyacrylamide gel electrophoresis.

*: Since several studies reported data from different regions, the total percentage exceeds 100%.

During the data analysis, heterogeneity was found in most of the analytical indicates; therefore, the random-effects model was applied throughout the analysis. When the outputs derived from all articles versus those from high-quality articles were compared, there was no difference in the temporal and spatial trends of rotavirus strain diversity; however, the magnitude of percentage showed slight inconsistencies. Thus, only analyses using all publications are presented hereafter.

### Diversity of rotavirus strains by region and by year

Overall, 22,112 and 10,660 rotavirus samples were examined for G and P types, respectively, from the 93 studies. In the past 19 years (1994–2012), the most common G types were G1 (39·5%), G3 (35·6%), and G2 (1·3%), followed by G9 (0·1%), but not G4 (<0·01%). For the P type, P[8] (54·6%) was predominant, followed by P[4] (11·1%) and P6 (0·1%) (**Tables 2** **and** **3**). The most common G-P combinations were G3P[8] (32·1%) and G1P[8] (24·5%), followed by G2P[6] (13·2%) and G2P[4] (10·1%) (**Table 4**).

**Table 2:** Distribution of common G types among children <5 years old with rotaviral diarrhea in China, 1994–2012.

Classification (number of studies)	Pooled percentage of G types among children with rotaviral diarrhea % (95% CI)					
	G1	G2	G3	G4	G9	G untyped
*By Year (Countrywide)*						
Before 2000 (n = 17)	74·3(71·6–77·1)	6·1(5·1–7·1)	4·7(3·8–5·6)	0·0(0·0–0·1)	0·0	0·4(0·1–0·7)
After 2000 (n = 81)	21·3(20·4–22·3)	0·9(0·8–1·1)	45·2(40·5–49·8)	0·0(0·0–0·1)	0·2(0·1–0·2)	10·9(9·8–11·9)
Overall (n = 93)	39·5(34·7–44·3)	1·3(1·1–1·5)	35·6(31·0–40·3)	0·0(0·0–0·1)	0·1(0·0–0·1)	6·7(6·1–7·3)
*By region and year*						
North						
Before 2000 (n = 12)	70·0(66·3–73·8)	6·6(5·2–7·9)	33·2(30·3–36·2)	0·0(0·0–0·1)	0·0(0·0–0·1)	0·5(0·1–0·9)
After 2000 (n = 41)	20·0(18·8–21·3)	1·2(0·9–1·5)	43·0(37·4–48·6)	0·0(0·0–0·1)	0·3(0·2–0·4)	10·2(8·8–11·5)
Overall (n = 49)	34·6(32·9–36·2)	1·7(1·4–2·0)	33·2(30·3–36·2)	0·0(0·0–0·1)	0·1(0·0–0·2)	6·1(5·3–6·8)
South						
Before 2000 (n = 8)	79·3(72·5–86·2)	6·8(4·8–8·9)	1·5(0·8–2·3)	0·0(−0·1–0·2)	0·0(0·0–0·1)	0·3(0–0·5)
After 2000 (n = 42)	24·2(22·5–26·0)	0·6(0·4–0·8)	47·6(37·4–57·9)	0·1(0·0–0·1)	0·1(0·0–0·2)	11·9(10·0–13·8)
Overall (n = 49)	39·9(29·3–45·5)	0·9(0·7–1·2)	37·4(29·3–45·5)	0·1(0·0–0·1)	0·1(0·0–0·1)	7·7(6·7–8·8)

**Table 3:** Distribution of common P types among children <5 years old with rotaviral diarrhea in China, 1994–2012.

Classification (number of studies)	Pooled percentage of P types among children with rotaviral diarrhea % (95% CI)			
	P4	P6	P8	P untypable
*By Year(Countrywide)*				
Before 2000 (n = 7)	8·2(6·4–10·1)	0·3(0·1–0·7)	49·9(36·6–66·2)	7·6(5·2–10·0)
After 2000 (n = 55)	8·7(8·0–9·5)	0·0(0·0–0·1)	55·3(48·5–62·1)	20·9(18·8–23·1)
Overall (n = 61)	11·1(10·2–12·0)	0·1(0·0–0·2)	54·6(48·7–60·6)	18·3(16·5–20·1)
*By region and year*				
North				
Before 2000 (n = 5)	17·2(10·2–24·2)	0·1(0·2–0·4)	53·1(29·0–77·2)	7·1(4·2–10·0)
After 2000 (n = 29)	7·4(6·4–8·3)	0·1(0·0–0·1)	56·4(48·4–64·3)	22·7(19·2–26·2)
Overall (n = 33)	7·3(6·5–8·1)	0·1(0·0–0·1)	55·4(42·7–68·1)	19·1(16·6–21·7)
South				
Before 2000 (n = 4)	6·6(4·0–9·3)	9·7(4·3–15·1)	42·9(34·1–51·7)	9·7(4·2–15·2)
After 2000 (n = 27)	11·3(10·0–12·7)	0·0(0·0–0·0)	53·2(37·0–69·3)	19·8(16·6–23·1)
Overall (n = 31)	0·6(0·4–0·8)	0·0(0·0–0·0)	51·4(37·52–65·3)	18·0(15·2–20·9)

**Table 4:** Distribution of common G-P combinations among children <5 years old with rotaviral diarrhea in China, 1994–2012.

G-P combination	Pooled percentage of G-P types %(95% CI) [number of studies]		
	North	South	Countrywide
G1P4	2·7(1·9–3·8)[9]	9·7(5·5–13·9)[11]	5·1(3·5–6·7)[20]
G1P8	28·5(19·0–38·1)[12]	21·6(15·9–27·2)[18]	24·5(19·9–29·2)[30]
G2P4	10·6(6·8–14·3)[13]	9·9(5·8–14·0)[10]	10·1(7·7–12·5)[22]
G2P6	13·2(3·5–22·8)[1]	-[0]	13·2(3·5–22·8)[1]
G3P4	5·2(3·0–7·4)[14]	6·6(4·3–8·9)[12]	5·7(4·2–7·2)[26]
G3P8	35·1(24·8–45·5)[14]	29·0(19·0–39·0)[17]	32·1(25·0–39·2)[31]

To determine the temporal trends of rotavirus strain distribution, we divided the time span at the year 2000, because before 2000, genotype G1 was the predominant strain and accounted for 74·3% of rotavirus infections, followed by G2 (6·1%) and G3 (4·7%). However after 2000, G3 (45·2%) became the predominant strain, followed by G1 (21·3%). Notably, G9 (0·2%), which was first identified in Yunnan province in 1998, increased in the most recent period (**Table 2**). In comparison, P types remained consistent across time periods. P[8] was always the predominant strain followed by P[4]. These top two strains were responsible for around 60% of rotavirus infection in each time period (**Table 3**).

There was good agreement on the spatial trends of rotavirus strain distribution between the North and the South of China. Similar to the trends countrywide, G3 replaced G1 and became the most prevalent strain in both regions. A slight difference was that more G9 strains were identified in the North after 2000 (**Table 2**). No differences in the distribution of P types was found by region (**Table 3**).

Among the less-common strains, G5, G8, P[9], and P[10] were notable. G5 emerged in 2003 in Hebei province. G8 mixed with G1 was first reported in Guanzhou city in 1994 and thereafter was identified in Beijing, Wuhan, Hunan, and Gansu provinces and reached a prevalence of 2% in some instances. Besides P[8], P[4], and P[6], P[9] and P[10] were relatively common strains during each time period. Comparatively, P[9] strains were identified more frequently in the South, and P[10] strains were more frequent in the North (**Table S1**).

## Discussion

In the most recent global systematic review, human serotypes G1, G2, G3, G4, and G9 were responsible for ∼90% of rotavirus infection among children less than 5 years of age [13]. A similar distribution of G types, as well as the temporal trends of rotavirus infection in China was obtained from our comprehensive systematic review, which covered studies over a 19-year time span. The prevalence of the G1 serotype, which accounted for >70% of rotavirus infection before 2000, decreased to ∼20% after 2000. In contrast, the number of identified G3 strains increased from 4·7% to 45·2% during the same period, and G9 emerged and reached a zenith of 19·2% in 2005 in the Xinjiang Autonomous Region [14]. Regardless of the fluctuation of G and P types overall, serotypes G1, G2, and G3 accounted for ∼80% of circulating strains in China, while the genotypes P[8] and P[4] were predominant consistently during that time period and represented ∼70% of rotavirus infections.

Currently it is not clear whether serotype-specific immunity plays an important role in protection against rotaviral gastroenteritis. However, the monovalent serotype G6 vaccine RIT 4237 was highly effective in locations where G1 was the predominant serotype, though homotypic immunity was found to be no more significant than heterotypic immunity in a multicenter study of RRV-TV in the USA. This results indicated the development of vaccine candidates should be based on human-animal reassortants containing the common serotype of human strains [15]. Two vaccine candidates have been developed in China. One, developed by Lanzhou Vaccine Institute, is a human-ovine reassortant vaccine containing human G2, G3, G4, and ovine P[12]. This human-ovine reassortant vaccine is being tested for efficacy in a large-scale phase III trial that recruited 12,000 infants less than 6 months old. Another candidate, which expresses human virus VP7 from serotypes G1, G2, G3, G4, G8, and G9, and VP4 (P[5]) from a bovine strain, is being developed by the Wuhan Vaccine Institute and is ready to enter clinical trials. With regard to serotype distribution, all those vaccine candidates could provide satisfactory protection of Chinese children, due to the broad coverage of prevalent serotypes in China.

Globally, a remarkable increase in the number of G8 strains in the African region was observed in recent year compared to the review article published in 1998 [13], [16], along with its identification in European countries [17], South Korea [18], India [19], Australia [20], and Asian countries. Although it is currently an unusual strain in China, since its emergence in Guangzhou city in 1994 [21], it has been identified in several provinces in the North and the South and even reached a prevalence of 2% over the past two decades [22]–[25]. Because G8 is presently circulating through China, it would make sense to add G8 in the construction of new vaccines. Despite the concern that might arise regarding the potential of G8 spreading, a study of RotaTaq demonstrated low incidence and low concentration of shedding when it was administered to infants [26]–[28]. These findings suggest that the vaccine strain is much less well adapted to growth at the infant intestinal mucosal surface, thus horizontal transmission among infants would be unlikely. Besides serotype, genotype might also play an important role in protection. Unfortunately, however, neither vaccine candidate included the most prevalent human virus VP4 (P[8]), which might render it less efficacious.

There are several limitations with our systematic review. First, not all studies aimed to identify the full spectrum of G and P types. Most studies focused on the common G and P types, namely G1–G4, and P[4], P[6], P[8], P[9], and P[10]. The unusual G and P types, such as G8 and G12 probably were misclassified or untypable, and thus we did not get full insight into the rotavirus strains circulating in mainland China. Notably, the prevalence of G and P untypable strains increased after 2000. Second, although the diversity of circulating rotavirus strains is very broad across the country, the virulence of each strain might not be equal. Unfortunately, we were not able to associate the severity of illness with the genotype of the corresponding strains, due to a paucity of data. Finally, differences between EIA- and PCR-based serotyping and genotyping might introduce heterogeneity and bias the accuracy of magnitude, rather than the trends.

In summary, continuing national epidemiological surveillance studies of the circulating strains of rotavirus will be vital to promoting future vaccine successes and to interpreting vaccine failures.

## Supporting Information

Checklist S1 PRISMA checklist.
(DOC)

Table S1 Unusual G-P types of rotavirus strains derived from diarrhea of children <5 years old in China, 1994–2012.
(DOCX)
