# Supplementary material for: Diversity of Rotavirus Strains Causing Diarrhea in <5 Years Old Chinese Children: A Systematic Review
Source: PLoS One. 2014 Jan 8;9(1):e84699. doi: 10.1371/journal.pone.0084699 (PMC3885581; doi:10.1371/journal.pone.0084699)
Supplement: Table S1 — Unusual G-P types of rotavirus strains derived from diarrhea of children <5 years old in China, 1994–2012. (DOCX) [file pone.0084699.s002.docx]

**Table S1.** Unusual G-P types of rotavirus strains derived from diarrhea of children <5 years old in China, 1994-2012

| Type | Region | Study period | No. typed | Positive | |
| --- | --- | --- | --- | --- | --- |
|  |  |  |  | No. | % |
| G Serotype | | | | | |
| G5 | Hebei[1] | 2007.1-2007.12 | 432 | 1 | 0.2 |
|  | Hebei[2] | 2003.8-2007.7 | 596 | 2 | 0.3 |
| G8 | Hunan[3] | 2009-2010 | 173 | 4 | 2.3 |
|  | Gansu[4] | 2009.1-2009.12 | 159 | 1 | 0.6 |
|  | Beijing[5] | 1995.1-1995.12 | 45 | 1 | 2.2 |
| P Genotype | | | | | |
| P[1] | Henan & Hebei[6] | 1989-1990 | 70 | 55 | 78.6 |
| P[2] | Henan & Hebei[6] | 1989-1990 | 70 | 15 | 21.4 |
| P[9] | Congqing[7] | 2008.10-2009.2 | 147 | 1 | 0.7 |
|  | Hebei[8] | 2006.1-2006.12 | 62 | 1 | 1.6 |
|  | Tianjin[9] | 2007.12-2009.12 | 133 | 3 | 2.3 |
|  | Hunan[3] | 2009-2010 | 173 | 1 | 0.6 |
|  | Shanghai[10] | 2005.10-2006.1 | 68 | 1 | 1.5 |
|  | Guangdong[11] | 2008.11-2009.2 | 64 | 1 | 1.6 |
|  | Guangdong[11] | 2007.11-2008.2 | 54 | 3 | 5.6 |
|  | Hebei[12] | 2006.1-2006.12 | 73 | 1 | 1.4 |
|  | Yunnan[13] | 1998.5-2001.4 | 58 | 1 | 1.7 |
|  | Guangdong[14] | 1998-1999 | 14 | 2 | 14.3 |
|  | Yunnan[14] | 1998-1999 | 16 | 1 | 6.3 |
|  | Sichuan[14] | 1998-1999 | 6 | 2 | 33.3 |
|  | Shandong[14] | 1998-1999 | 21 | 1 | 4.8 |
|  | Zhejiang[14] | 1998-1999 | 9 | 2 | 22.2 |
|  | Jilin[15] | 2008 | 235 | 1 | 0.4 |
|  | Jilin[15] | 1999-2000 | 47 | 1 | 2.1 |
|  | Hebei[16] | 1999-2000 | 54 | 1 | 1.9 |
|  | Zhejiang[16] | 1999-2000 | 12 | 2 | 16.7 |
|  | Yunnan[16] | 1999-2000 | 50 | 1 | 2.0 |
|  | Guangdong[16] | 1999-2000 | 30 | 2 | 6.7 |
|  | Hebei[2] | 2003.8-2007.7 | 369 | 1 | 0.3 |
|  | Hebei[17] | 2006.1-2007.12 | 404 | 1 | 0.3 |
| P[10] | Congqing[7] | 2008.10-2009.2 | 147 | 1 | 0.7 |
|  | Hebei[8] | 2006.1-2006.12 | 62 | 1 | 1.6 |
|  | Jilin[18] | 2007.10-2008.11 | 417 | 8 | 1.9 |
|  | Hunan[3] | 2009-2010 | 173 | 5 | 2.9 |
|  | Guangdong[11] | 2007.11-2008.2 | 54 | 1 | 1.9 |
|  | Hebei[12] | 2006.1-2006.12 | 73 | 1 | 1.4 |
|  | Jilin[15] | 2008 | 235 | 3 | 1.3 |
|  | Hebei[19] | 1990-1992 | 102 | 1 | 0.9 |
|  | Jilin[2] | 2003.8-2007.7 | 497 | 1 | 0.2 |
|  | Hebei[2] | 2003.8-2007.7 | 369 | 1 | 0.3 |
|  | Hebei[17] | 2006.1-2007.12 | 404 | 1 | 0.3 |
| P-G combinations | | | | | |
| G1P[1] | Henan & Hebei[6] | 1989-1990 | 70 | 52 | 74.3 |
| G1P[6] | Guangxi[20] | 2007-2008 | 88 | 7 | 7.9 |
|  | Guangdong[11] | 2007.11-2008.2 | 54 | 6 | 11.1 |
|  | Guangdong[21] | 1998.11-2001.1 | 25 | 1 | 4.0 |
|  | Yunan[13] | 1998.5-2001.4 | 41 | 2 | 4.9 |
|  | Gansu[4] | 2009.1-2009.12 | 116 | 3 | 2.6 |
| G1P[9] | Guangdong[21] | 1998.11-2001.1 | 25 | 2 | 8.0 |
| G1P[10] | Hebei[19] | 1990-1992 | 132 | 1 | 0.9 |
| G2P[2] | Henan & Hebei[6] | 1989-1990 | 70 | 13 | 18.6 |
| G2P[8] | Jiangsu[22] | 2009.7-2010.6 | 113 | 4 | 3.5 |
|  | Gansu[23] | 2004.7-2005.6 | 94 | 3 | 3.2 |
|  | Jiangsu & Anhui[24] | 2001.9-2002.12 | 86 | 3 | 3.5 |
|  | Shanghai[10] | 2005.10-2006.1 | 68 | 1 | 1.5 |
| G2P[10] | Hebei[25] | 2006.1-2006.12 | 58 | 1 | 1.7 |
|  | Gansu[26] | 2004.7-2005.6 | 94 | 3 | 3.2 |
| G3P[1] | Henan & Hebei[6] | 1989-1990 | 70 | 2 | 2.9 |
| G3P[6] | Hebei[27] | 1999.7-2003.6 | 100 | 4 | 4.0 |
|  | Gansu[4] | 2009.1-2009.12 | 116 | 17 | 14.7 |
| G3P[9] | Shanghai[10] | 2005.10-2006.1 | 68 | 1 | 1.5 |
| G4P[1] | Henan & Hebei[4] | 1989-1990 | 71 | 1 | 1.4 |
| G4P[4] | Henan[28] | 2009.1-2009.12 | 56 | 1 | 1.8 |
| G4P[6] | Hebei[27] | 1999.7-2003.6 | 100 | 5 | 5.0 |
| G4P[8] | Shanghai[29] | 2001 | 87 | 7 | 8.1 |
| G9P[4] | Guangdong[30] | 2009.1-2009.12 | 132 | 1 | 0.8 |
| G9P[6] | Yunan[13] | 1998.5-2001.4 | 41 | 5 | 12.2 |
|  | Xinjiang[31] | 2005 | 52 | 3 | 5.8 |
| G9P[8] | Xinjiang[31] | 2005 | 52 | 7 | 13.5 |
|  | Shanghai[32] | 2006.11-2008.1 | 437 | 10 | 2.3 |
|  | Hebei[33] | 2006.11-2007.11 | 45 | 2 | 4.4 |
|  | Yunan[13] | 1998.5-2001.4 | 41 | 2 | 4.9 |
|  | Gansu[34] | 2001-2006 | 262 | 5 | 1.9 |
|  | Jiangsu[35] | 2001.9-2002.8 | 63 | 1 | 1.6 |
|  | Henan[28] | 2009.1-2009.12 | 56 | 1 | 1.8 |
|  | Hebei & Beijing[36] | 2001.8-2003.7 | 470 | 12 | 2.6 |
|  | Hubei[37] | 2005.10-2006.9 | 91 | 2 | 2.2 |
|  | Guangdong[30] | 2009.1-2009.12 | 132 | 1 | 0.8 |
| G1G3P[4] | Neimenggu[38] | 2008.1-2010.11 | 50 | 3 | 6.0 |
|  | Jiangsu[39] | 2001.9-2003.6 | 69 | 3 | 4.4 |
|  | Guangdong[30] | 2009.1-2009.12 | 132 | 15 | 11.4 |
|  | Heilongjiang[40] | 2007.1-2007.12 | 70 | 2 | 2.9 |
| G1G3P[8] | Zhejiang[41] | 2009.11-2010.4 | 153 | 19 | 12.4 |
|  | Hubei[37] | 2005.10-2006.9 | 91 | 8 | 8.8 |
|  | Neimenggu[42] | 2008.6-2009.5 | 125 | 20 | 16.0 |
|  | Shanghai[32] | 2006.11-2008.1 | 437 | 12 | 2.8 |
|  | Guangdong[25] | 2009.1-2009.12 | 45 | 1 | 2.2 |
|  | Heilongjiang[40] | 2007.1-2007.12 | 70 | 3 | 4.3 |
|  | Gansu[34] | 2001-2006 | 262 | 4 | 1.5 |
| G1G4P[8] | Jiangsu[35] | 2001.9-2002.8 | 63 | 10 | 15.9 |
| G2G3P[4] | Zhejiang[41] | 2009.11-2010.4 | 153 | 1 | 0.7 |
|  | Gansu[26] | 2004.7-2005.6 | 94 | 7 | 7.5 |
|  | Gansu[34] | 2001-2006 | 262 | 10 | 3.8 |
|  | Neimenggu[42] | 2008.6-2009.5 | 125 | 1 | 0.8 |
| G2G3P[8] | Shanghai[10] | 2005.10-2006.1 | 68 | 3 | 4.4 |
| G3G4P[8] | Shanghai[29] | 2002 | 100 | 14 | 14.0 |
|  | Jiangsu[43] | 2001.9-2003.8 | 80 | 6 | 7.5 |
|  | Neimenggu[38] | 2008.1-2010.11 | 50 | 5 | 10.0 |
|  | Shanghai[32] | 2006.11-2008.1 | 437 | 4 | 0.9 |
|  | Jiangsu[39] | 2001.9-2003.6 | 69 | 7 | 0.1 |
| G1G2P[4]P[8] | Hebei[19] | 1990-1992 | 102 | 2 | 1.9 |
| G1G3P[6]P[8] | Hebei[19] | 1990-1992 | 102 | 1 | 0.9 |

**List of Included Studies**

1. Yang SH, Wang H, Liu N, Cui SX, Li DD, et al. (2009) Molecular epidemiology of rotavirus among children under 5 years old hospitalized for diarrhea in China. Zhong Hua Shi Yan He Lin Chuang Bing Du Xue Za Zhi 23: 168-170.
2. Duan ZJ, Liu N, Yang SH, Zhang J, Sun LW, et al. (2009) Hospital-Based Surveillance of Rotavirus Diarrhea in the People's Republic of China, August 2003-July 2007. J Infect Dis 200 Suppl 1 S167-173.
3. Li JH, Zhou SF, Liu YZ, Deng ZH, Huang W, et al. (2012) Etiological Study on Viral Diarrhea Among Infants and Young Children in Surveillance Hospitals of Hunan Province from 2009 to 2010. Shi Yong Yu Fang Yi Xue 19: 337-341.
4. Wei KF, Yang JJ, Liu XF, Liu JD, Chen JH, et al. (2010) Analysis of children rotavirus diarrhea in Lanzhou in 2009. Zhong Guo Wei Sheng Jian Yan Za Zhi 20: 2025-2027.
5. Qiao H, Nilsson M, Abreu ER, Hedlund KO, Johansen K, et al. (1999) Viral diarrhea in children in Beijing, China. J Med Virol 57: 390-396.
6. Fang ZY, Ji SJ, Qin SM, Zhao XF, Wu D (1994) Serotyping/Genotyping of group A rotavirus by use of PCR method. Bing Du Xue Bao 10: 316-321.
7. Liao Y, Chen JH, Zhu CM, Xu HM, Liu ZY (2010) Molecular epidemiologic characterization and related data analysis of rotavirus in infants of Chongqing. Di San Jun Yi Da Xue Xue Bao 32: 77-80.
8. Yu QL, Liu L, Liu JS, Tang JY (2008) A etiology Surveillance of Virus Diarrhea in Hebei Province in 2006. Zhong Guo Quan Ke Yi Xue 11: 479-481.
9. Zhang ZL, Zhang Y, Li JM, Gao L, Liu H, et al. (2011) The study on rotavirus infection status among patients with diarrhea in Tianjin from 2007 to 2009. Zhong Guo Re Dai Yi Xue 11: 930-932.
10. Lin Y, Li M, Ma HL, Jiang XF ( 2007) Detection of genotype of group A rotavirus by nested-polymerase chain reaction. Jian Yan Yi Xue 22: 377-379.
11. Zong WP, Xiang WL, Yu SY, Chen Q, Hu GF ( 2011) Molecular epidemiological study on the characteristics of rotavirus diarrhea during autumn and winter season in Guangzhou. Shi Yong Yi Xue Za Zhi 27: 682-684.
12. Yu QL, Liu JS, Han Z , Liu L, Tang JY, et al. (2007) Rotavirus genotype among infants with diarrhea in Hebei Province in 2006. Zhong Guo Wei Sheng Jian Yan Za Zhi 17: 2270-2272.
13. Zhang LJ, Du ZQ, Zhang Q, Kang HY, Zhen LS, et al. ( 2004) Rotavirus surveillance data from Kunming Children's Hospital, 1998-2001. Zhong Hua Liu Xing Bing Xue Za Zhi 21: 353-356.
14. Fang ZY, Qi J, Yang H, Wang CX, Ye Q, et al. (2001) Distribution of serotyping/genotyping of rotavirus strains derived from infants with diarrhea in China, 1998-1999. Bing Du Xue Bao 17: 17-23.
15. Zhang XJ, Li LH, Li WM, Liu Y, Zhao YL, et al. (2011) Study of Etiology and Clinical Data for Infants with Rotavirus Diarrhea in Changchun Province in 2008. Zhong Guo Yi Miao He Mian Yi 17: 59-61.
16. Fang ZY, Yang H, Qi J, Zhang J, Sun LW, et al. (2002) Diversity of rotavirus strains among children with acute diarrhea in China: 1998-2000 surveillance study. J Clin Microbiol 40: 1875-1878.
17. Li DD, Liu N, Yu JM, Zhang Q, Cui SX, et al. (2009) Molecular epidemiology of G9 rotavirus strains in children with diarrhoea hospitalized in Mainland China from January 2006 to December 2007. Vaccine 27 Suppl 5: F40-45.
18. Zhao Y, Qi YL, He SY ( 2011) Analysis of the infants virus diarrhea disease surevillance result in Jilin City between 2007 and 2008. Zhong Guo Shi Yan Zhen Duan Xue 15: 1878-1881.
19. Adhikary AK, Zhou Y, Kakizawa J, Numaga J, Akihara S, et al. (1998) Distribution of rotavirus VP4 genotype and VP7 serotype among Chinese children. Acta Paediatr Jpn 40: 641-643.
20. Chen MM, Zhou KJ, Mo KJ, Tan Y, Li H, et al. (2010) Molecular epidemiologic study of rotavirus diarrhea among children in Luocheng County, Guangxi in 2007~2008. Ying Yong Yu Fang Yi Xue 16: 75-77.
21. Xie JP, Fang ZY, Zhang Q, Lai GX, Zhu B, et al. ( 2004) The detection rate and serotype distribution of rotavirus strains from children with diarrhea in Guangzhou city, 1998-2001. Zhong Hua Shi Yan He Lin Chuang Bing Du Xue Za Zhi 18: 82.
22. Lin Q, Zhou JS, Lu F, Li XL, Li M, et al. (2011) Study on Clinical Characteristics and Molecular Epidemiology of Younger than 5 Years Old Children with Diarrhea Caused by Rotavirus Infection in Nanjing City in 2009－2010. Shi Yong Er Ke Ling Chuang Za Zhi 26: 1709-1711.
23. Zeng M, Zhu QR, Zhang Y, Li GH, Chen DM, et al. (2004) Molecular epidemiologic survey of rotaviruses from infants and children with diarrhea in Shanghai. Zhong Hua Er Ke Za Zhi 42: 10-15.
24. Song XB, Tao FB, Ding H, Wang P (2004) Clinical study on rotavirus serotype G and P patterns among children under 5 years old with acute diarrhea in Mananshan and Suzhou city. Lin Chuang Er Ke Za Zhi 22: 433-435.
25. Zhao DP, Hua L, Zhao MQ (2011) Molecular epidemiology of nosocomial rotavirus gastroenteritis among children in Guangzhou. Zhong Hua Yi Yuan Gan Ran Xue Za Zhi 21: 4248-4250.
26. Ye XH, Jing Y, Fang ZY, Sun YP, Xie HP, et al. (2006) Etiological study on viral diarrhea among children in Lanzhou, Gansu, from July 2004 through June 2005. Zhong Hua Liu Xing Bing Xue Za Zhi 27: 117-122.
27. Fang ZY, Zhang LJ, Zhang Q, Hu HK, Xie HP, et al. ( 2005) Study on rotavirus diarrhea among children in Lulong County, Hebei province, China. Bing Du Xue Bao 21: 21-26.
28. Jiao LY, Guo QH, Song ZS, Lu GJ ( 2010) Molecular Epidem lologic Characteristics ofRotavirus Diarrhea in Infants in Northern Henan. Shi Yong Er Ke Ling Chuang Za Zhi 25: 1199-1200.
29. Xu J, Sun JE, Ding YZ, Su LY, Yang Y (2007) Molecular epidemiology of group A rotavirus in 1450 hospitalized children in Shanghai, China,2001-2005. Zhong Guo Xun Zhen Er ke Za Zhi 2: 102-107.
30. Xie HP, Geng JM, Yu YJ, Wei YH, Lu EH, et al. (2011) Molecular Epidemiological Characteristics ofRotaviruses in Guangzhou,2009. Yu Fang Yi Xue Qing Bao Za Zhi 27: 773-776.
31. Yang XL, Matthijnssens J, Sun H, Muhamaiti J, Zhang B, et al. ( 2008) Temporal changes of rotavirus strain distribution in a city in the northwest of China, 1996-2005. Int J Infect Dis 12: e11-17.
32. Zeng M, Zhang Y, Zhu Q, Wang X, Yu H ( 2010) Clinical and molecular epidemiology of rotavirus in children with community-acquired and hospital-acquired diarrhea in Shanghai, China. Pediatr Infect Dis J 29: 177-180.
33. Liu L, Zhou JK, Zhao L, Li W, Wang XM ( 2008) Study about rotavirus infection among infants within 5 years in Shijiazhuang. Zhong Guo Wei Sheng Jian Yan Za Zhi 18: 1489-1491.
34. Jin Y, Ye XH, Fang ZY, Li YN, Yang XM, et al. (2008) Molecular epidemic features and variation of rotavirus among children with diarrhea in Lanzhou, China, 2001-2006. World J Pediatr 4: 197-201.
35. Shen H, Li H, Zhang J, Gu HY, Wang P ( 2003) Molecular epidemological study on rotavirus diarrhea in infants in Suzhou. Zhong Guo Gong Gong Wei Sheng 19: 1420-1421.
36. Fang ZY, Wang B, Kilgore P. E, Bresee J. S, Zhang LJ, et al. ( 2005) Sentinel hospital surveillance for rotavirus diarrhea in the People's Republic of China, August 2001-July 2003. J Infect Dis 192 Suppl 1: S94-99.
37. Yang J, Wang T, Wang Y, Lu B, Bai X, et al. (2007) Emergence of human rotavirus group a genotype G9 strains, Wuhan, China. Emerg Infect Dis 13: 1587-1589.
38. Bi XD, Mei H (2010) Epidemiological study on rotavirus diarrhea among children in Hohhot, 2008- 2010. Neimenggu Yi Xue Yuan Xue Bao 32: 110-112.
39. Tian JM, Xu L, Shen H, Jing H ( 2003) Clinical epidemiology study on rotavirus diarrhoea among children less than 5 years in Suzhou. Xiao Er Ji Jiu Yi Xue 10: 370-371.
40. Ren LN, Zhang BL, Liang Y, Huai QJ, Liu XH, et al. ( 2009) Molecular epidemiologic characteristics of rotaviruses from infants and children with diarrhea in Harbin. Zhong Guo Wei Sheng Jian Yan Za Zhi 19: 596-599.
41. Wang JW, Liu CX, Yang AP, Zhang Y, Liu Y, et al. ( 2011) G serotyping and P genotyping of rotavirus A isolated from children with diarrhea. Zhong Guo Gong Gong Wei Sheng 27: 296-298.
42. Yu Q, Li CY, Wang WR ( 2011) Epidemiological research of infantile diarrhea with rotavirus in Hohhot, 2008- 2009. Zhong Hua Ji Bing Kong Zhi Za Zhi 15: 52-55.
43. Wu QB, Gu HY, Tang WF, Jing H, Wang P (2005) Molecular epidemioloic characteristics of rotavirus diarrhea in Suzhou. Jiang Su Yi Yao 31: 99-101.
